# Supplementary material for: Causal Relationship between Aging and Anorexia Nervosa: A White-Matter-Microstructure-Mediated Mendelian Randomization Analysis
Source: Biomedicines. 2024 Aug 16;12(8):1874. doi: 10.3390/biomedicines12081874 (PMC11351342; doi:10.3390/biomedicines12081874)
Supplement: Supplementary file 1 [file biomedicines-12-01874-s001.zip › Supplementary Figure/Figure S8/Leaveoneout plot of MR analysis for TL to ubm-b-1519.pdf]

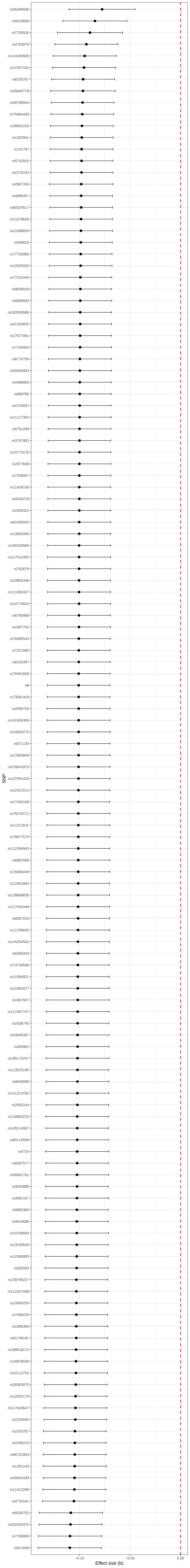
rs35446936
rs9419958
rs7705526
rs2763979
rs143190905
rs11557154
rs8105767
rs35640778
rs56799554
rs75664430
rs28502153
rs1332941
rs131797
rs5742915
rs2276182
rs2967355
rs4695407
rs80324517
rs11579626
rs11699829
rs939916
rs77732866
rs12925933
rs77231040
rs9600019
rs6669563
rs182059586
rs41304832
rs17677991
rs7164950
rs6776756
rs59409453
rs4498805
rs869785
rs4743037
rs11117354
rs6751209
rs3767952
rs10773176
rs2977608
rs7209057
rs11426156
rs4530278
rs1003322
rs61405042
rs13062095
rs150150565
rs117512405
rs762679
rs10805346
rs111950327
rs10774624
rs6790988
rs1907702
rs76065543
rs7221585
rs8102497
rs78491606
All
rs73581419
rs2056726
rs142426306
rs10840270
rs871134
rs17803849
rs376641875
rs137901416
rs12412214
rs17445108
rs76219171
rs11212631
rs79977579
rs112394943
rs6881568
rs76666449
rs12451892
rs139669835
rs117034449
rs6007020
rs11769630
rs144204502
rs6590343
rs73730598
rs11584821
rs11991877
rs1957937
rs117407747
rs2538745
rs10845387
rs450962
rs185174247
rs113525195
rs9940099
rs141214782
rs2555104
rs116863223
rs145114957
rs80116508
rs4724
rs6587577
rs56061761
rs3093888
rs3891167
rs9955360
rs4616688
rs10768683
rs13230646
rs12369950
rs932002
rs139795227
rs111527438
rs10905255
rs7099229
rs1985369
rs61748181
rs188918174
rs16978028
rs10112752
rs28363070
rs12932179
rs117630647
rs2230590
rs1023767
rs3785074
rs66731853
rs1291143
rs45604339
rs11412296
rs4731541
rs6536702
rs202034370
rs7790856
rs611646
SNP
-0.10
-0.05
0.00
Effect size (b)
